# Assumptions and conceptual contributions to improve the global assistive performance of actual wheelchairs, in order to enhance the users’ autonomy and quality of life

**Published:** 2012

**Authors:** I Andone, G Onose, V Avramescu, V Cardei, C Orăşanu

**Affiliations:** * Bagdasar Arseni - Teaching Emergency Hospital, Bucharest, Romania; ** “Carol Davila” University of Medicine and Pharmacy, Bucharest; *** SC ICTCM - Mechanical Engineering and Research Institute SA, Bucharest, Romania

**Keywords:** optimized “all in one” wheelchair, autonomy, Quality of Life (QoL), QoL = Quality of Life, CAD = Computer-Aided Design, CATIA = Computer Aided Three-dimensional Interactive Application, CAE = Computer-Aided Engineering, ADAMS = Advanced Data Management System, MMSE = Mini Mental State Examination, WhOM = Wheelchair Outcome Measure, SCI-FCS = Falls Concern Scale for people with Spinal Cord Injury, ADL = Activity of Daily Living, SCI = Spinal Cord Injury, RDI = Researcher Development Initiative

## Abstract

**Rationale**: Recovery of autonomy, in paralyzed/with severe disabilities patients, is one of the most difficult challenges for rehabilitation. Thus, an appropriate wheelchair is essential for this kind of people, both in daily lives, including work and social participation, and for quality of life (QoL).

**Objective:** The purpose of the study is to achieve a consistent improvement to the actual models of wheelchairs followed by validation through clinical trial of the optimized prototype, in order to enhance the users’ autonomy and QoL.

**Material and Results:** In the research activities and for establishing constructive and optimized functional solutions will be used, simulation of system operation techniques, based on software packages and Computer-Aided Design/ Engineering (CAD/ CAE) systems.

Validation, of the optimized wheelchair prototype, through clinical trial, requires a prospective study. The study will include a group of 30 patients, who will be investigated for a one-month period. The patients will complete, at the end of the study, a standardized questionnaire containing generic data and many items referring to the optimized wheelchair functions and to the autonomy of the users including in relation to their own expectations. We will also use the quantified evaluation scale of QoL, Wheelchair Outcome Measure (WhOM) and a Falls Concern Scale for people with Spinal Cord Injury (SCI-FCS).

**Discussion:** The wheelchair particularities that we pursue, and which are distinguished from the other models, is the fact that the wheelchair is powered, pliable and allows verticalization, hopefully at a price comparable or even lower than the current state of the art models (but none of them succeeded by now to fulfil all this three basic functions on a single “all in one” such device). Hence, if our optimized prototype will achieve technical and clinical validation, this will result in a significant enhancement of autonomy and QoL for the users.

## Introduction

Mobility plays an important role in the lives of almost all of the disabled - especially with Spinal Cord Injury (SCI) - persons and its limitation is affecting the ability to participate in nearly all the activities of the daily living [**1**]. Having a proper wheelchair may improve QoL of this people, giving them the independence they need and desire to live with more freedom. Some important roles of wheelchairs are to increase functionality, to improve independence, and to allow a person to lead a successful life at home and in the community [**2,3**].

Before explaining the importance of an optimised wheelchair in these people’s lives, it is necessary to mention some brief historical data of the wheelchair followed by the description of the wheelchair and its components.

**Fig. 1:**
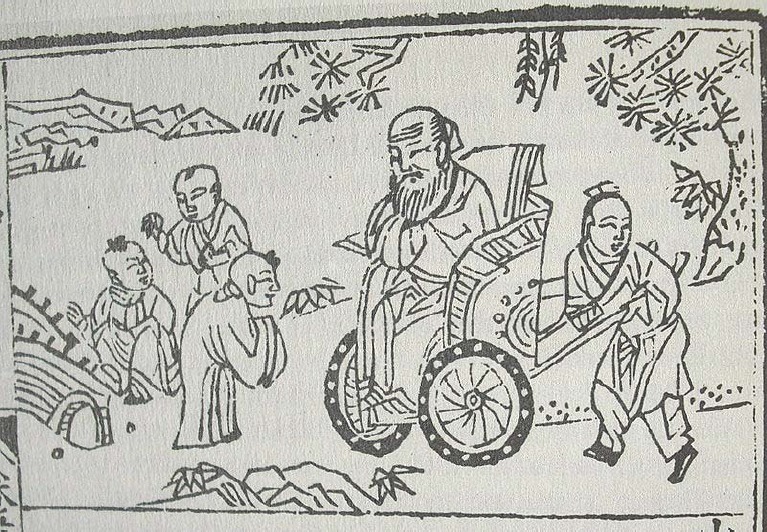
Depiction of Confucius in a wheelchair, dating to ca. 1680 [**5**]

The appearance of the wheelchair implied first the invention, separately, of its essential components: wheels - approximately 3500 years BC - and then the chair, attested around 2900 BC, originated in eastern Mediterranean basin. The earliest record of wheelchairs was found on an inscription on a stone slate in China, which dates back to the 6th century (**Fig. 1**) [**4**].

**Fig. 2:**
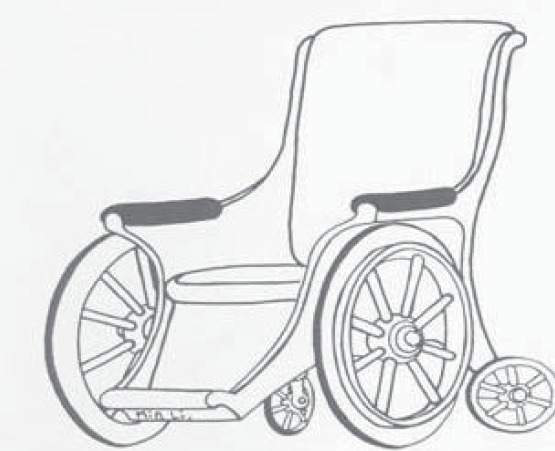
Pliable wooden wheelchair dating from American Civil War [**8**]

The first record of combining wheels to furniture was an image of a wheeled child’s bed on Greek vase 530 BC [**5**]. Later times, relate the using of this technology in Europe, during German Renaissance. The invalid carriage or Bath Chair seems to date from around 1760 [**6**]. In the eighteenth century, a wheelchair with two large wheels in front and a small one behind (extremely unstable) appeared. In the nineteenth century smaller and lighter, wheelchairs, made of wood, with bicycle-like wheels and with special circles for manual drive have been developed. During the American Civil War, a non-pliable wooden wheelchair, with two large wheels in front and two small wheels behind appeared (**Fig. 2**) [**7,8**].

The first pliable wooden wheelchair seems to have been manufactured in the USA by an assistive devices factory owner (having a daughter with a severe motor deficit). Harry Jennings and his disabled friend Herbert Everest, both mechanical engineers, who invented the first lightweight, steel, pliable wheelchair [**9**], did the next big improvement in the wheelchair domain over seven decades ago.

Unfortunately, this was also the last major improvement to this type of assistive device, until the appearance of the engines applied on wheels, thereby, preserving the pliability (in the last around 10-15 years). Advancement and update of wheelchair, continued with the development of various technologies and materials [**7,8**]

**Fig 3.**
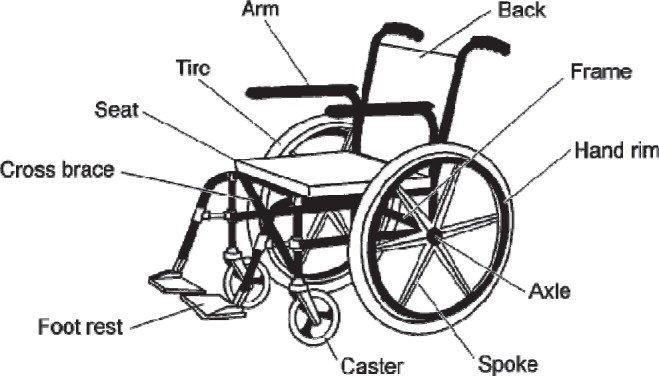
Description of the wheelchair parts [**10**]

The main structural components of a wheelchair (**Fig. 3**) [**10**] are: tires, wheels, hand’s circles (manual propelled), chairs, backrests (sitting support/ insurance/ assistance system), legs support devices and armrests and respectively, cushions (to be mentioned that although the cushions are not technically intrinsic wheelchair components, their medically importance – especially for pressure sore prevention is huge and in this respect recent models of wheelchairs and cushions with alternating pressure/ air flow are considered including in constructive formulae for mutual matching [**11**] **-** a synoptic overview of the wheelchair cushions’ main aspects is presented in **Table 2**) [**12**].

**Table 2.** Synoptic overview of the wheelchair cushions’ main aspects [**11**]

***Materials***	***Applications***	***Advantages***	***Disadvantages***
foam	general application	Good stability, cheep	pressure is not optimally distributed
dressed and contoured foam	general application	excellent stability, easy to clean, durable	produces heat, expensive
(filling/ core of) gel	general application	dispense pressure, easy to clean, no heat production	expensive
foam with gel	when is required a good pressure distribution	good relief for pressure distribution, easy to clean, resistance	expensive, produces heat
air cells	when there is need of optimal pressure distribution	excellent pressure distribution, easy to clean, no heat production	Expensive, not offer optimal stability

The frame is the base for all other wheelchair components and can be composed of aluminum, steel, plastic/ composite materials, wood [**10**] is stable, and can be rigid or pliable (as improvements at this part of wheelchair level, our optimised prototype will be: **ultra-pliable** and with **variable geometry**, allowing **verticalization** and as light as possible but strong enough to withstand intended use).

We intend to improve some other parts of the current wheelchair too, using original constructive solutions, such as: the back rest equiped with control options allowing back adequacy position; arm rest to be telescopic, foot rests to be able to slide in sagital plan, anti-overturning sistems, and others which by very easy to understand reason can not be yet detalied.

**Fig. 4:**
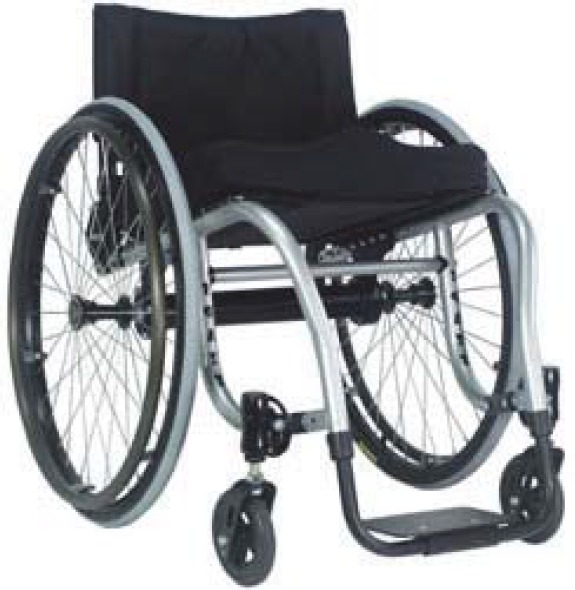
Ultra light wheelchair [**10**]

**Fig. 5:**
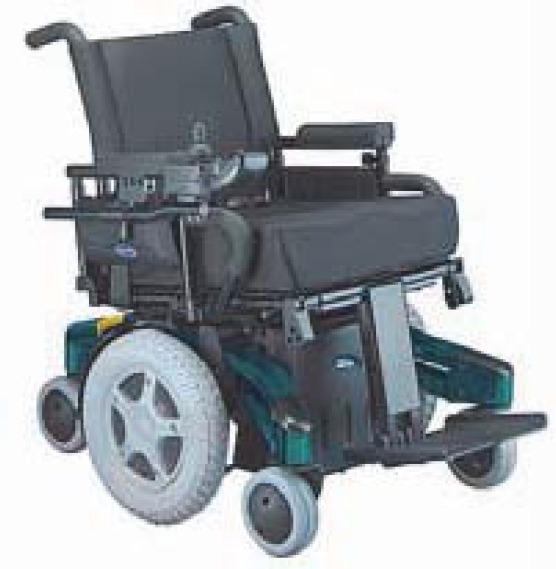
Electric wheelchair [**10**]

The types of wheelchairs available today can be classified according **to age of use**: adult/pediatric, **weight:** heavy/medium/light/ultra-light (**Fig. 4**) **propelling modality**: manual powered/motorized/electric (**Fig. 5**), **the frame design**: pliable/non-pliable, with verticalization possibilities/to convert into a stretcher/tilting/non-tilting, (from) metal/composite materials, etc. (**Table 1**) [**12**].

**Table 1.** Synoptic overview of the types of wheelchairs, with their main characteristics and indications for use [**11**]

***Type of wheelchair***	***Characteristics***
Rigid frame	Non-pliable, used in institutions and in sport
“X” frame	Pliable, ordinary wheelchair
Powered by user	The user propels the wheelchair
Powered by an assistant	Assistant push wheelchair, this can have four small wheels. Used in medical / medico-social institutions
Motorized	Powered by an electric motor
Standard weight	Usual configuration
Light/ ultra-light	For special individual activities, we believe that it should become standard
Wheelchair used for sport	For specific activities; and it has lower/ below back unto the usual one and has no armrests, to facilitate upper body activities
For adults	Usual configuration
For children	Available in different versions at different sizes, possibly with some adjustable components
With verticalization facilities	It facilitates the users standing
Inclinable	For patients with spasticity and / or pressure sores and / or lung problems
It can turn into a stretcher	For patients with orthostatic hypotension and / or pressure sores

**Fig. 6:**
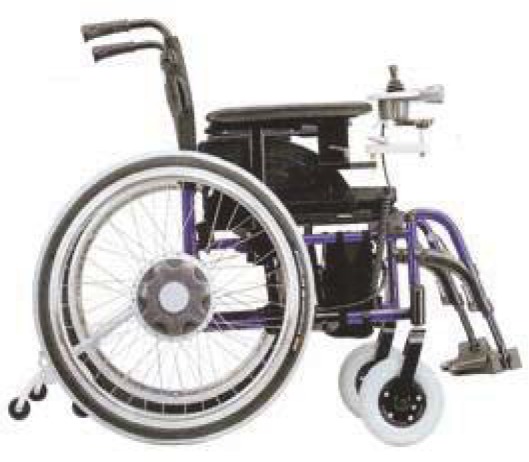
Old type of pliable motorised wheelchair with joystick [**13**]

**Fig. 7:**
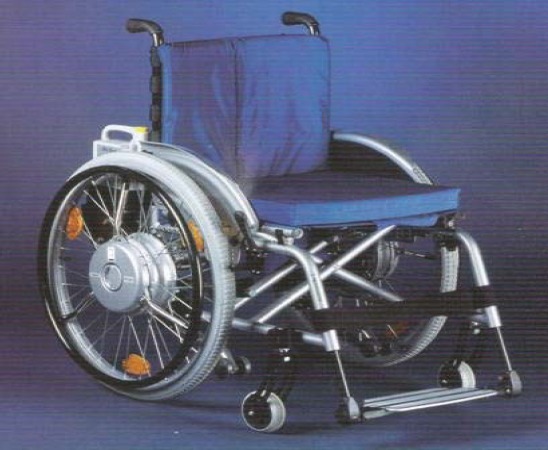
New type of wheelchair without joystick/ remote control [**13**]

Recently, a new form of control and motor’ action in motorised wheelchair category has been developed consisting in connecting the concentric circles for manual (auto) propulsion, directly to the engine, at the homologue wheel [**12**]. The user does not need to do anything but to initiate the propelling: thus, he/ she has during linear movement, both hands free; ones he/she has given an initially impulse to the circles of the wheels, the motorised wheels equipped with sensors will continue the acceleration until the user will interfere braking both circles on the wheels symmetrically (to reduce speed/stop) or unilaterally to negotiate curves. Some older models of the pliable wheelchairs present a joystick (**Fig. 6**) but this newer one has no more need for joystick or remote control (**Fig. 7**) during rolling [**13**].

**Fig. 8:**
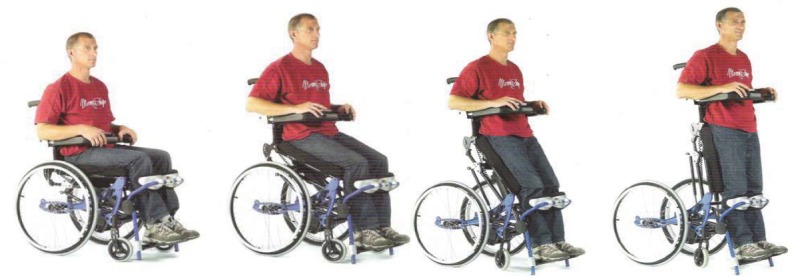
A stand up type of wheelchair [**12**]

The types of wheelchair, which can help the patient stand up (**Fig. 8**), are using the user’s mechanical force of the arms or electrical energy in this respect. Regular and sustained standing up, contribute to prevention/combating the pressure sores and also osteoporosis, improvement of the urinary – gravitational – drainage and bowel transit, ascending – including at real means – at psychological horizon. The paralyzed user is able, through such a device, to reach by himself, for example, racks, shelves, etc., that have been "banned" until this assistive facilities have been developed [**14,15**]. Thus, the standing up device is an important component of the wheelchair that contributes to the improvement of the users’ autonomy and QoL and because of that, it is one of our principal constructive solutions to achieve an optimised prototype of the wheelchair.

There are only two well-known producers for a device of this kind, worldwide and the price is very high/prohibitive for many potential users. Such type of wheelchairs (even in their simplest versions - with verticalization through mechanical self actuation) are not pliable and are not provided with motorized travel opportunities in this respect, i.e. they are not equipped with engines mounted on the wheels level.

*Wheelchair prescribing purposes*: Patients, who use a wheelchair for the first time, often ask the practitioner to prescribe one with as many functions as possible, but they usually manage to find that a wheelchair with too many options is more difficult to handle. Therefore, we can say that no single wheelchair answer the needs of all users and the wheelchair must be adapted to the physical needs of the assisted persons, environmental demands and availability of resources. Proper evaluation is necessary to identify the right wheelchair’s adjustments and solutions [**10**].Because a wheelchair is often used for several hours per day and during all activities, to have the proper one can make the difference between dependence and reasonable, relative independence. In this respect, the most important prescribing purposes are [**16**]:

**-** *maximizing the mobility *and *its effectiveness *above the environment, regarding the achievement of the mainstreamed, global goal - represented by the autonomy. From this perspective, the wheelchair must have stable functions with low power consumption and with minimal assistance from others.

- *prevention/minimizing deformation and/or injuries*. Wheelchair must contribute to pressure sores, muscle contractures, deposturation, prophylaxis and any other pathogenic events related to its use.

**-** *Projection of a healthy and as much as it is possible, attractive image, of the body *[**16**].

**Fig. 9:**
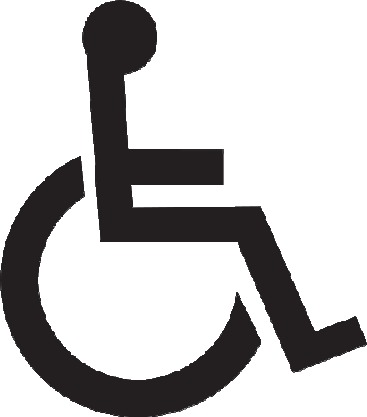
International symbol of wheelchair [**5**]

The *population groups *with maximal *potential to benefit from an optimised wheelchair *with the purpose of keeping/regaining their own functional autonomy and thus, their QoL, are the most complex and markedly disabling conditions, such as: tetra/paraplegia, severe hemiplegia and also, some of the “oldest old” group (characterized by large polipathology and highest levels of polidisabilities, especially of: neurological/locomotion, cardiologic and respiratory, kinds) [**17**]. Therefore, it is not just a coincidence that the wheelchair image is disability and handicap’s international symbol (**Fig. 9**) [**5**].

## Methods

From the descriptions analyses of some representative wheelchairs made by a variety of companies, the necessity of some improvement results, both on conceptual and functional plan, for which, the most adequate functional and constructive solutions have not been found yet. We consider that a motorized, optimised wheelchair must fulfil at least six essential performance conditions, in order to be really functional and useful for assisted persons:

1. sitting support / safe assistive system (including anti-overturning advanced capabilities);
2. to remain pliable so the wheelchair can be placed in a car trunk;
3. through motorization (with all required subassemblies) neither weight nor volume of the wheelchair should increase significantly;
4. electrical loading batteries proceedings and respectively, the engine consumption, to allow the user's functional autonomy for at least 3 hours;
5. the actual price paid by an insured user for achieving such a device not to be prohibitive;
6. to be able to fulfil a multitask functions – mainly including verticalization (within the afore mentioned “all in one” concept).

An additional purpose is the attempt to realize within the “all in one” prospective, another function of the wheelchair, narrowing for penetrating in tight spaces (such as couloirs within aircrafts) without necessitating the patient to be temporally removed from his/her wheelchair.

In the research activities and, for establishing constructive and optimized functional solutions will be used, for choice, simulation of system operation techniques based on software packages (Solid Works). For 3D modelling, advanced CAD systems such as CATIA (Computer Aided Three-dimensional Interactive Application), Unigraphics, SolidWorks, SolidEdge, Solid Designer, etc., will be used. For kinematics mechanisms, simulation CAE systems will be used: ADAMS (Advanced Data Management System) and WorkingModel and for analysis of static, dynamic and thermal action, CAE systems: ANSYS, COSMOS, warped, etc. These simulation techniques on the computer are very important for a better understanding of all the movements made by the device and all the forces that exert on the wheelchair – that can be thus represented -, being in this way an economic modality to prevent not working solutions before their construction.

For the validation of the optimised wheelchair, a prospective study that will include a group of 30 patients will be used. They will be investigated for a one-month period.

*Criteria for admission/exclusion of patients in the study group*: paraplegics patients; twenty to sixty years old persons (fortunately for the researcher and unfortunately for them, most of this patients are young and so uncomplicated); with no pressure sores; with a level of motivation (we will give these people a preliminary a mini questionnaire to complete as an annex of the informed consent, to evaluate the level of motivation); material aspects; patients with at least an average level of cognitive capacity tested through the Folstein questionnaire for Mini Mental State Examination (MMSE) [**18**].

Primary data acquisition will be based on a *standardised questionnaire *completed by the patient at the end of the wheelchair using period (consisting on **demographic data**: name - hidden/ codified -, gender, age, marital status, the type of affection that causes the infirmity and **data referring to the wheelchair**: type of wheelchair used before the study, the ramp approaching mode, comfort, stability in outdoor conditions, concrete situations inaccessible with a wheelchair, the attitude in adverse weather conditions, problems of rubbing certain parts of the body with the wheelchair seat, back, etc.; time and effort required to mount the gambier stabilizers, technical events, response and safety/ balance in standing position, urinary self catheterization – hypothetically radically improved through verticalization -, autonomy duration in wheelchair use) the answers being quantified as ordinal variables.

The elements which will also be evaluated: QoL (using a quantified scale for the assessment of QoL); level of participation (assessed by WHOM questionnaire, the only tool available for the determination of the activities performed with wheelchair [**19**]); the patients’ level of satisfaction; and the risk of falls in patients with spinal lesions (using Falls Concern Scale for people with spinal cord injury (SCI-FCS) **Table 3**) [**20**].

**Table 3.** Falls Concern Scale for people with spinal cord injury (SCI-FCS) [**20**]

***SCI-FCS activity***	***Not at all concerned *1**	***Somewhat concerned *2**	***Fairly concerned *3**	***Very concerned *4**
Getting dressed or undressed	1	2	3	4
Moving around the bed (including sitting up)	1	2	3	4
Inserting enema or toileting	1	2	3	4
Washing or showering self	1	2	3	4
Transferring on/off a commode or toilet	1	2	3	4
Transferring in/out of bed	1	2	3	4
Transferring in/out of a car	1	2	3	4
Reaching for high objects (e.g. reaching to a high shelf/lift button)	1	2	3	4
Picking objects up from the floor (e.g. clothes, pet bowl)	1	2	3	4
Cooking or food preparation (e.g. making a sandwich)	1	2	3	4
Pushing wheelchair on flat ground	1	2	3	4
Pushing wheelchair on an uneven surface (e.g. rocky ground)	1	2	3	4
Pushing wheelchair up/down gutters or curbs	1	2	3	4
Pushing wheelchair up/down a slope	1	2	3	4
Shopping	1	2	3	4
Lifting heavy objects across body (e.g. shopping bags)	1	2	3	4

Our standardized, afore exposed questionnaire, together with the international assessment tools for QoL, level of participation, respectively of satisfactions and risk of falls – all filled in with the specific data/answers collected from the patients in our study group - will form the basis of complex documentation for patenting.

## Discussion and Conclusion

**Fig. 10:**
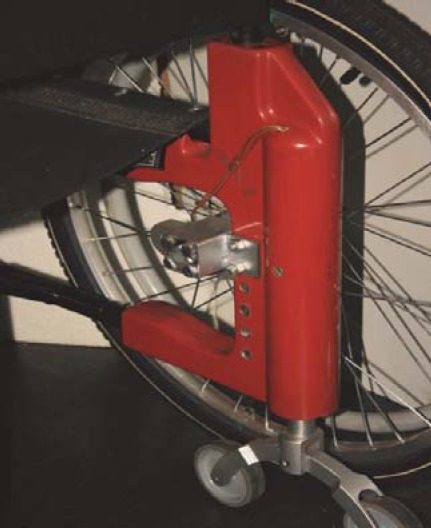
Lifting device [**21**]

**Fig. 11:**
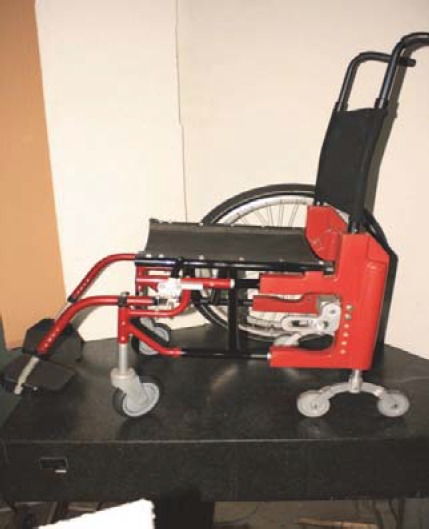
An integrated conceptual model of wheelchair materialised through the achievement of some of its structure components [**21**]

It is important to mention that during 2006-2008, within a RDI (Researcher Development Initiative) Project, through a large multidisciplinary consortium, the authors have already achieved the first experimental model of an improved wheelchair, able to propel by motors on the wheels and to verticalize, but, which is not yet reaching all the afore exposed intended items, especially because of its weight. As a preliminary result, we present two figures of the integrated conceptual model of wheelchair materialised through the achievement of some of its structure components (**Fig. 10,11**) [**21**].

To make our new experimental model more functional and also trying to add on it some new optimizations, we propose a set of complex improvements (presented above) that could lead to the achievement of a consistent optimized model of wheelchair, able to profoundly enhance the users’ autonomy.

For the achievement of this prototype, we must also take into account: new mechanical design approaches, in order to satisfy the needs of the user; to lower the energy consumption; to reduce the cost and weight and respectively, to enhance the reliability – the latter will be the main issue to be considered, so that the optimised wheelchair could be put into the practical use in the near future.

The target of the study is therefore of great actuality and social importance and the solutions for increasing global assistive performances contain novelty elements and are real assumptions for the improvement of the autonomy and QoL of the assisted persons. To accomplish these goals we intend to use the latest achievements of technology. However, in order to easily understand the reasons, more details will be offered after the project completion, validation of the optimised wheelchair and achievement of intellectual property protection. Therefore, they will be issued in future related published works.
